# Effects of altered contractile environment on muscle shape change in the human triceps surae

**DOI:** 10.1242/jeb.248118

**Published:** 2024-12-02

**Authors:** Nicole Y. Kelp, Kylie Tucker, François Hug, Taylor J. M. Dick

**Affiliations:** ^1^ School of Biomedical Sciences, University of Queensland, St Lucia, 4072 Queensland, Australia; ^2^ Université Côte d'Azur, LAMHESS, Nice, France

**Keywords:** Electromyography, Gearing, Muscle force, Pennation angle, Ultrasonography

## Abstract

Skeletal muscles change shape when they contract. Current insights into the effects of shape change on muscle function have primarily come from experiments on isolated muscles operating at maximal activation levels. However, when muscles contract and change shape, the forces they apply onto surrounding muscles will also change. The impact of an altered contractile environment (i.e. mechanical behaviour of surrounding muscle) on muscle shape change remains unknown. To address this, we altered the mechanical contributions of the human gastrocnemii during isometric plantarflexion contractions [via changing knee angle] and determined if there were associated changes in how the muscles of the triceps surae bulged in thickness during a ramped contraction. We combined B-mode ultrasound imaging with surface electromyography to quantify the neuromechanical contributions of the medial gastrocnemius (MG), lateral gastrocnemius (MG) and soleus (SOL) muscles during isometric plantarflexion contractions. Our results demonstrate that at the same SOL activity levels, altering knee angle had no influence on the magnitude of muscle shape change (thickness) in the triceps surae muscles. We observed high levels of inter-individual variability in muscle bulging patterns, particularly in the knee flexed position, suggesting a complex relationship between muscle bulging and activation strategies in the triceps surae, which may be related to differences in muscle mechanical properties between participants or across muscles. Our findings highlight the dynamics of *in vivo* bulging interactions among muscles within the triceps surae and provide insights for future investigations into the impact of altered contractile environments on three-dimensional muscle deformations and force production.

## Teaser

**Summary:** Altering the mechanical behaviour of surrounding muscles has limited influence on changes in skeletal muscle shape, suggesting a complex relationship between muscle bulging and activation strategies in synergists.

## INTRODUCTION

In a concentric contraction, muscle fascicles shorten as a result of actin–myosin cross bridging, generating force to shorten the whole muscle. Given that muscles are isovolumetric, they must bulge radially as they shorten (Baskin and Paolini, 1967). In pennate muscles, where fascicles are arranged at an angle relative to the muscle's line of action, muscle fascicle shortening is accompanied by changes in pennation angle. This amplifies the shortening of the whole muscle and allows muscle fascicles to shorten at a slower velocity than the whole muscle, a term known as gearing (Azizi et al., 2008). Previous *in vitro* and *in situ* studies on isolated muscles have shown that when a muscle bulges in thickness (defined as the distance between the superficial and deep aponeuroses), this permits more fascicle rotation, whereas when a muscle is constrained from bulging in thickness, this limits fascicle rotation, and thus gearing (Azizi et al., 2008; Brainerd and Azizi, 2005). However, a series of *in vivo* studies have demonstrated that muscles belonging to the same anatomical group may change shape in different ways, behave differently and display varying degrees of fascicle rotation during both submaximal and maximal contractions (Kelp et al., 2021; Maganaris et al., 1998; Randhawa et al., 2013; Wakeling et al., 2011). Together, these studies suggest a complex interplay between individual muscle bulging and fascicle rotation across muscles that belong to the same anatomical group.

Variations in muscle shape change and fascicle behaviour influence how a muscle generates force, allowing it to adapt to different mechanical demands and to optimize its performance across a range of activities (Azizi et al., 2008; Dick and Wakeling, 2017; Randhawa et al., 2013; Tijs et al., 2021; Wakeling et al., 2011). Muscle architecture, internal muscle properties and the external environment, including the mechanical behaviour of adjacent muscles and potential compressive factors, possess the capacity to constrain or modify the dynamic changes in muscle shape and gearing. These constraints, whether imposed (or modified) by ageing (Holt et al., 2016; Narici et al., 2021), disease (Gao and Zhang, 2008), disuse (de Boer et al., 2008) or an altered contractile environment (Ryan et al., 2019), can limit a muscle's capacity to deform, ultimately constraining fascicle shortening and limiting contractile forces.

The mechanical interactions between muscles within a synergist group likely influence their bulging patterns. Muscles, such as the triceps surae, are packaged tightly within a fascial compartment and are constrained by not only the surrounding connective tissue, but by the muscle activity and bulging behaviours of other muscles within that compartment. Skeletal muscles are therefore mechanically connected to surrounding structures and these connective tissue interactions enable the transmission of force between muscles (Maas et al., 2001; Maas and Sandercock, 2010). There is also a growing body of evidence that altering a muscle's ability to deform during a contraction influences its mechanical behaviour (Azizi et al., 2017; Ryan et al., 2019; Siebert et al., 2018, 2014). For example, when polypropylene sleeves were used to physically constrain a muscle's bulging, muscle shortening and work output both decreased (Azizi et al., 2017). Siebert and colleagues have demonstrated that when a muscle is transversely loaded, there is a reduction in maximum isometric force and rate of torque development in both rat (Siebert et al., 2014) and human muscles (Siebert et al., 2018). Similarly, when the human medial gastrocnemius (MG) is transversely loaded during contractions, muscle bulging and fascicle rotation both decrease (Ryan et al., 2019). Together, these studies provide evidence that variations in shape change and fibre rotation among muscles may arise, at least in part, from the contractile environment in which the muscle operates. Herein, we refer to the contractile environment as the mechanical state of the surrounding muscles.

The three heads of the triceps surae exhibit differences in their shape change and activation patterns across muscles, individuals and tasks (Ahn et al., 2011; Crouzier et al., 2018; Hug et al., 2021; Lay et al., 2007). The MG and LG are bi-articular, functioning as both ankle plantar flexors and knee flexors, while the SOL is monoarticular, crossing only at the ankle joint. Knee flexion reduces the capacity of the MG and LG to produce force (Gordon et al., 1966) as the muscle fascicles are at shorter lengths (Lauber et al., 2014). Varying the knee angle alters the distribution of activation between the three muscles of the triceps surae, with flexed positions leading to reduced muscle activity (Hebert-Losier and Holmberg, 2013) and motor unit firing (Lauber et al., 2014) in the gastrocnemii but not in the SOL. This means that we can use changes in knee angle to alter the external contractile environment (i.e. mechanical state of surrounding muscles) in which the SOL contracts within, without directly influencing the SOL.

Here, we explore how alterations in the contractile environment, induced by changes in knee angle and therefore the neuromechanical contribution of the gastrocnemii, influences muscle shape changes in the triceps surae. Specifically, we aimed to determine if there are associated changes in bulging patterns of the SOL, MG and LG when the mechanical contribution of surrounding muscles is altered by changing knee angle during isometric plantarflexion contractions. To achieve this, we combined B-mode ultrasound to simultaneously image the MG, LG and SOL with surface electromyography to quantify the neuromechanical contributions of each muscle during isometric plantarflexion contractions at different knee angles. SOL muscle activity was matched for all conditions. We hypothesized that for the same SOL activity level, the SOL would bulge more in thickness when the surrounding gastrocnemii muscles are less active (i.e. at more flexed knee angles), owing to a reduction in their force-generating capacity, and are therefore less likely to bulge.

## MATERIALS AND METHODS

### Participants

Seventeen volunteers participated in this study. All participants were between 18 and 35 years with no lower limb injury or pain that kept them from work or sport in the prior 6 months. Written informed consent was provided by each participant, based on the experimental protocols approved by the University of Queensland's Human Research Ethics Committee (2022/HE001089). Out of the 17 participants, four were excluded from data analysis due to poor ultrasound image quality of the SOL and two were excluded because of their inability to accurately follow the ramp biofeedback during the experiment. Eleven participants (5 females, 6 males; 26±5 years; 69.8±19.3 kg; 175.6±9 cm) were included in the analysis (Table 1).

**Table 1.**
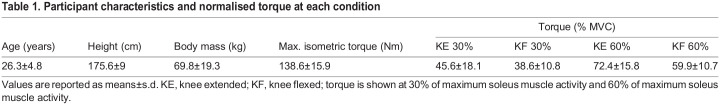
Participant characteristics and normalised torque at each condition

### Experimental protocol

**Fig. 1.**
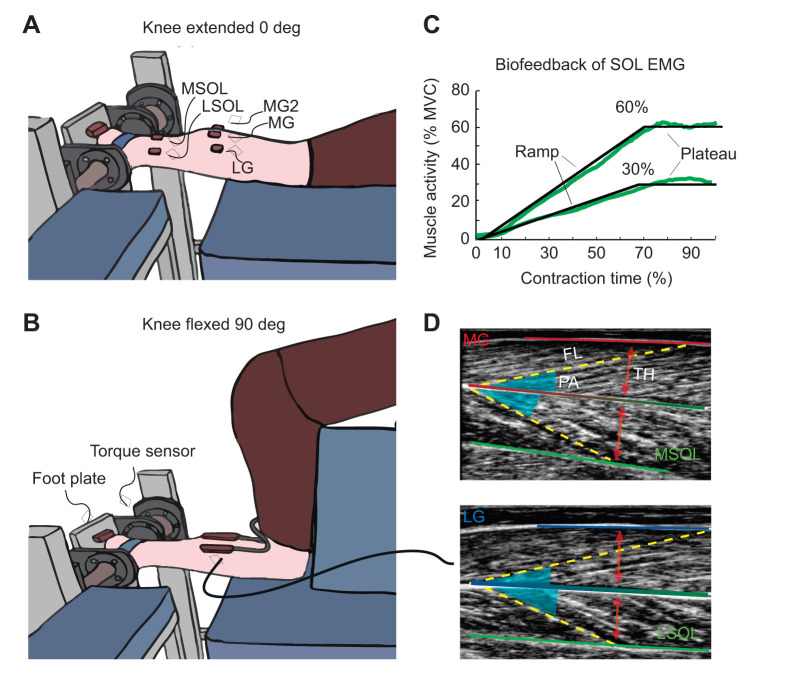
**Experimental set-up.** Participants (*n*=11) performed isometric plantarflexion contractions with the knee extended (0 deg) (A) and the knee flexed (90 deg) (B) with their foot strapped into a custom-built isometric dynamometer. Bi-polar surface electromyography (EMG) electrodes were placed on the lateral gastrocnemius (LG), the medial gastrocnemius (MG and MG2), and the medial and lateral soleus (MSOL and LSOL). (C) EMG signals (black line: biofeedback signal; green line: EMG signal) from MSOL and LSOL were averaged (mean) and normalized to the maximum EMG recorded for each muscle during a maximum voluntary contraction (MVC). The root mean square of the average normalized signal over a 500 ms moving window was provided as a biofeedback signal to the participant. Participants performed ramped contractions at 30% and 60% of their maximum SOL EMG at each knee angle. (B,D) A two-probe ultrasound setup was used to simultaneously measure the MG, LG and SOL during each contraction. Fascicle length (FL), pennation angle (PA) and muscle thickness (TH) were measured for each muscle.

Participants were positioned prone with their ankle at 90 deg and foot securely fastened to the foot plate of a custom-made ankle dynamometer (Fig. 1). They performed maximum voluntary isometric plantarflexion contractions (MVCs) a minimum of three times, and until the maximum torque was within 10% of the previous trial, with a 2 min rest between contractions. Participants then performed isometric plantarflexion contractions at 30% and 60% of the maximum SOL muscle activity recorded during the MVC, herein referred to as 30% and 60% SOL EMG, during two conditions, knee extended at 0 deg and knee flexed at 90 deg. We controlled for the level of SOL EMG activity under different ‘environmental’ conditions (i.e. knee angles) to ensure that we isolated the altered mechanical environment effect on changes in muscle behaviour (i.e. thickness), rather than the effects of altered SOL muscle activity. The filtered and smoothed SOL EMG values were provided as biofeedback on a computer monitor placed in front of the participant. A ramp-plateau protocol was used in which participants were asked to follow a pre-determined trace overlaid on their SOL EMG biofeedback signal. This trace consisted of a 3 s ramp to their specified target, and a hold at the plateau for 3 s before returning to rest (Fig. 1C). Participants were given two trials at each condition and contraction level to familiarize themselves with the biofeedback protocol. The participants were instructed to follow the pre-determined trace as close as possible. A trial was repeated if the participant began the ramp early, had excessive fluctuations in their ramp or hold, or over-/under-shot the target hold more than 5%. This was determined visually during each trial. Experimental trials were then pseudo-randomized, such that the participant would randomly start with their knees flexed or knees extended, then perform three trials each in random order at 30% and 60% SOL EMG, before switching to the other knee angle position and repeating the 30% and 60% SOL EMG contractions in random order. The 30% SOL EMG contractions had a 20 s rest and the 60% SOL EMG contractions had a 40 s rest between each trial within each knee angle condition and a 1 min rest when knee angle was changed. During the knee flexed conditions, a goniometer was used to position the knee at the required angle, while participants leaned forward over a wooden box that supported their torso, with their thighs and hips resting against the box (Fig. 1B). Triceps surae muscle activity was recorded for three trials at each condition for a total of 12 trials (see Electromyography section below). Some EMG electrodes were then removed (owing to space restrictions on the leg) and an ultrasound probe was placed over each gastrocnemii muscle (two probes total) to simultaneously image the MG, LG and underlying SOL (Fig. 1D) (see ultrasound section below). The ramped contraction protocol was repeated while capturing ultrasound data. Trials were repeated.

### Data acquisition and analysis

#### Electromyography

Muscle activity of the MG, LG, medial and lateral SOL was measured using wireless bipolar surface EMG (10 mm inter-electrode distance; Trigno Delsys Inc., Natick, MA, USA) (Fig. 1A). Electrodes were placed on the muscle bellies and aligned with the muscle fascicle direction, as determined using B-mode ultrasound. The skin was prepared by shaving, lightly abrading (Nuprep Skin Prep Gel, Weaver and Company, Colorado, USA) and cleansing with alcohol to minimize the skin–electrode impedance. Electrodes were attached to the skin with double-sided tape and elastic bandages were used to secure the electrodes. The use of elastic bandages was repeated for both the EMG and ultrasound trials to provide similar amounts of compression between the trials. During the EMG trials, two electrodes were placed on the MG. One was positioned at the optimal location specified by SENIAM guidelines (seniam.org), and over the area at which the ultrasound transducer would later be placed, while the other MG electrode was positioned medially. The medially placed electrode allowed for the collection of both MG and SOL muscle activity during the ultrasound trials, which was then used to verify that the MG maintained similar levels of activity between EMG and ultrasound trials at matched conditions. Space restrictions on the skin directly above the LG muscle belly did not allow for the recording of LG muscle activity during the ultrasound trials. EMG signals and a torque signal from the ankle dynamometer as well as synchronization triggers from the ultrasound devices were acquired in Spike2 at 2048 Hz (V10, CED Ltd, Cambridge, UK). Biofeedback of SOL muscle activity was given in real time as an average of the lateral and medial SOL, normalized to that obtained during maximal voluntary contractions (MVCs) and processed using a moving root mean square with a time constant of 500 ms (Fig. 1C).

Raw EMG and torque signals were exported to and analysed in MATLAB (version 9.5.0; R2018b, MathWorks Inc., Natick, MA, USA). Raw EMG signals were bandpass filtered between 20 and 500 Hz and visually inspected for noise or artifact. The root mean square (500 ms moving window) was processed to create an EMG envelope. Torque signals were lowpass filtered at 12 Hz. Across the three MVC trials, peak EMG amplitude for each muscle was determined and the maximum value was used to normalize the corresponding EMG for each of the experimental trials. Maximum torque was also determined from the MVC trials and was used to normalize the measured torque values across the different experimental conditions. EMG and torque signals were synchronized to the onset of the ramp ending midway through the plateau region where torque level remained constant (3 s. ramp and 1.5 s plateau) then interpolated to 100 data points. SOL activity was determined as the average of normalized signals from the medial and lateral SOL EMG. EMG and torque values were averaged across the three trials performed at each condition. To compare muscle activity during the hold between muscles and conditions, the average amplitude of each muscle was calculated during the isometric plateau whereby force was constant (i.e. the average of the EMG signals between 80 and 90% of the contraction time). To compare temporal changes in muscle activity between muscles and conditions, average time-varying amplitudes for each muscle during each condition were calculated across the entire contraction time from onset of the ramp to midway through the plateau region.

#### B-mode ultrasound

A dual probe B-mode ultrasound configuration (LV8-5L60N-2, 60 mm, linear, 8 MHz, Telemed, Vilnius, Lithuania) was used to simultaneously image the MG and LG, as well as the SOL, deep to at least one of the gastrocnemii muscles. The SOL was imaged under either the MG (*n*=7) or LG (*n*=4), depending on which position allowed for the SOL fascicles to remain in the imaging plane during the entire contraction; therefore, only one value is presented for the SOL. Ultrasound probes were positioned over the same regions as the EMG electrodes and aligned in the plane of the muscle fascicles. This meant that for the MG and LG, the ultrasound was placed in the middle of the muscle belly; however, this position would vary for the SOL, with positioning being more proximal or distal depending on the placement of the probe over the gastrocnemii. Custom-built 3D-printed ultrasound probe holders and elastic bandages were used to secure the probes in place and limit probe movement during the contractions. The ultrasound systems (ArtUS Ext-1H, REV: C1S, Telemed, Vilnius, Lithuania) were externally triggered at 120 Hz from a data acquisition board (CED 1401, Cambridge Electronic Design Ltd., Cambridge, UK), initiated by the manufacturer's Spike2 software 1 s before the onset of the ramp protocol and ultrasound images were recorded in Echowave II software (Telemed, Vilnius, Lithuania).

Fascicle length, pennation angle and muscle thickness were determined from the ultrasound images using semi-automated ultrasound tracking software [UltraTrack v.5.3 adapted by Doguet and Hauraix (2019) from the original UltraTrack v.4.2 by Lichtwarck and Farris (2016)], which uses an affine optic flow tracking algorithm (Cronin et al., 2011) to track tissues throughout each frame of the ultrasound video. Within each video, regions of interest and tissues were manually selected to include and identify the superficial and deep aponeurosis, as well as two fascicles within each video (Fig. 1D). Careful visualization of fascicle and aponeurosis movement was performed to ensure accurate tracking, with manual corrections made as necessary. Time-varying fascicle length and pennation angle were exported as the average of the two fascicles tracked in each video. All fascicle length, pennation angle and muscle thickness measures were exported to Mathematica (v.11.3, Wolfram Research, Inc., Champaign, IL, USA) for further analysis. Time-varying fascicle length, pennation angle and muscle thickness values were lowpass filtered at 12 Hz with changes in fascicle length and muscle thickness normalized to resting values. Resting muscle architecture was measured before the contraction at each condition (i.e. knee extended and knee flexed) when the muscle was inactive. Change in pennation angle is expressed in deg. Ultrasound data was synchronized to the same onset and offset times as the EMG data and interpolated to 100 data points. Change in muscle fascicle length, muscle thickness and pennation angle were determined during the isometric plateau whereby torque was constant (i.e. the average of the signal between 80 and 90% of the contraction time). The maximum change in muscle thickness, regardless of the muscle getting thinner (negative values) or thicker (positive values) was also calculated as the difference in the distance between the superficial and deep aponeuroses.

### Statistical analysis

A Welch's *t*-test was used to examine differences in torque between the knee extended and knee flexed conditions. Linear mixed effects models (lme.R function from the nlme package; CRAN.R-project.org/package=nlme) with participant included as a random factor, were used to determine the effect of knee angle and muscle (fixed factors) on (1) muscle activity, (2) resting muscle architecture (fascicle length, pennation angle and thickness) and (3) the changes in fascicle length, pennation angle and thickness (dependent variables) (R v.3.4.3; r-project.org). Tukey's *post hoc* tests were used to assess any significant interactions and differences between muscles (glht.R function from the multcomp package; Hothorn et al., 2008). All statistical results from the full linear mixed-effects models and Tukey's *post hoc* tests can be found in Table S1.

One-dimensional statistical parametric mapping (SPM) was used to perform a two-tailed *t*-test to compare time-varying changes in SOL muscle thickness during the knee extended and knee flexed conditions, at each contraction level (30% and 60% SOL EMG) (spm1d, open source MATLAB v.M.0.4.1; Pataky, 2010). A two-way repeated measures ANOVA using one-dimensional SPM was used to determine the effect of knee angle and muscle (fixed factors) on the time-varying changes in muscle thickness (MG, LG, and SOL, dependent factors). For all tests, differences were considered significant at *P*<0.05.

## RESULTS

### Influence of knee angle on muscle activity and resting architecture

**Fig. 2.**
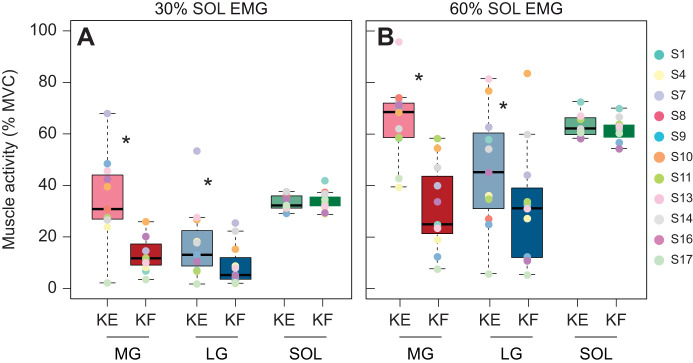
**Medial and lateral gastrocnemii muscle activity decrease in the knee-flexed compared with knee-extended condition.** Normalized muscle activity (% maximum voluntary contraction, MVC) of the medial gastrocnemius (MG; red), lateral gastrocnemius (LG; blue), and soleus (SOL; green) during the plateau region of the ramped isometric plantarflexion contraction at 30% (A) and 60% (B) of maximum SOL EMG for each knee angle, knee extended (KE; lighter colours) and knee flexed (KF; darker colours). Box and whisker plots indicate the median (black line) with boxes representing the first and third quartiles (quartile range). Whiskers represent the 95% CIs. Individual data for each participant (S) is shown via different colour points (*n*=11). Linear mixed effects models were used to assess significance. Asterisks (*) denote significant difference (*P*>0.05) between knee extension and knee flexion.

Participants maintained the same SOL EMG level during both the knee-extended and the knee-flexed conditions (30% SOL EMG: *P*=0.841; 60% SOL EMG: *P*=0.070; Fig. 2). As expected, gastrocnemii muscle activity was lower when the knee was flexed in both the 30% SOL EMG (MG, *P*<0.001; LG, *P*=0.013) and 60% SOL EMG (MG, *P*<0.001; LG, *P*=0.024) conditions (Fig. 2). Specifically, MG activity was 21.6±18.2% lower at 30% SOL EMG and 33.5±23.5% lower at 60% SOL EMG in the knee-flexed compared with the knee-extended condition. While the LG was less active than the MG, LG activity was also 8.3±16.7% lower in the knee-flexed condition at 30% and 14.0±33.3% at 60% SOL EMG, compared with the knee-extended condition. There was a large amount of variability in MG and LG normalized EMG amplitudes between participants across conditions, indicated by the range and interquartile range (IQR) in the box plots of Fig. 2. The time-varying muscle activity patterns across conditions are presented in the Results. Ankle plantarflexion torque was lower when the knee was flexed compared with extended at 60% SOL EMG condition (*P*<0.001), but not during the 30% SOL EMG condition (Table 1).

MG and LG fascicle lengths at rest (i.e. no contraction) were shorter in the knee-flexed compared with the extended position by 13.9±11.2 mm (*P*<0.001) and 6.6±14.1 mm (*P*<0.001), respectively (Table 2). SOL fascicle lengths were shorter in the knee-flexed position compared with the knee-extended position by 2.8±10.7 mm (*P*=0.007). Pennation angle was 3.5±4.6 deg. greater in the flexed compared with extended position in the MG (*P*=0.005), with no difference between positions for the LG (*P*=0.116). SOL pennation angle was greater in the knee-extended condition by 1.3±4.3 deg (*P*=0.004). The MG and LG were 1.9±1.9 mm and 2.0±2.9 mm narrower, respectively, in the knee-flexed position (both *P*<0.001), with no difference in SOL resting muscle thickness (*P*=0.989) between knee angles (Table 2).

**Table 2.**
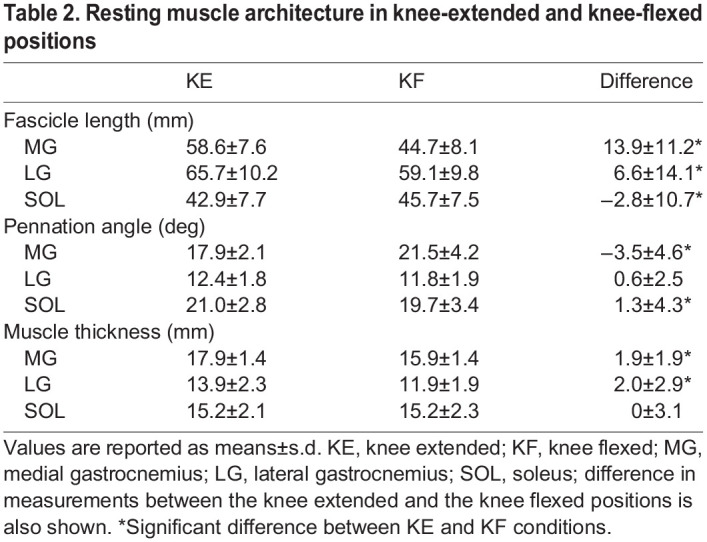
Resting muscle architecture in knee-extended and knee-flexed positions

### Effect of knee angle on triceps surae shape changes during a contraction

#### Muscle thickness

**Fig. 3.**
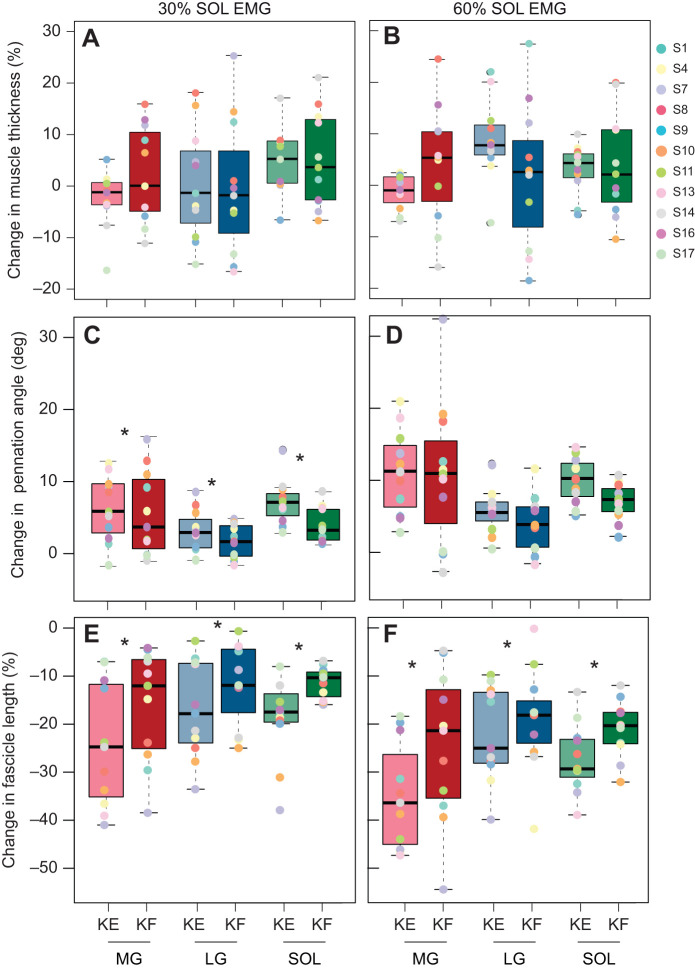
**Muscle shape and architecture change at different knee angles.** Muscle thickness changes were not different between the knee-extended and knee-flexed conditions. However, fascicle rotation (at 30%) and shortening (both 30% and 60%) were lower when the knee was flexed. The change in muscle thickness (normalized to resting muscle thickness at each condition) (A,B), pennation angle in deg (C,D) and fascicle length (normalized to resting fascicle length at each condition) (E,F) for the medial gastrocnemius (MG; red), lateral gastrocnemius (LG; blue) and soleus (SOL; green) during the plateau region of the ramped isometric plantarflexion contractions at 30% (A,C,E) and 60% (B,D,F) of maximum SOL EMG for each knee angle with knee extended (KE; lighter colours) and knee flexed (KF; darker colours). Linear mixed effects models were used to assess significance. Asterisks (*) denote significant difference (*P*>0.05) between knee extension and knee flexion.

There was no effect of knee angle on the magnitude of muscle thickness change between rest and the plateau region of the isometric contraction (30% SOL EMG, *P*=0.610; 60% SOL EMG, *P*=0.903) nor any differences in thickness changes between muscles during the plateau (30% SOL EMG, *P*=0.083; 60% SOL EMG, *P*=0.332) (Fig. 3A,B). However, there was a large amount of variability in muscle thickness changes between participants across all muscles, particularly when comparing changes in muscle thickness in the knee extended versus knee flexed position (denoted by the range and IQR in the boxplots of Fig. 3A,B).

#### Pennation angle

There was a main effect of knee angle and muscle on the change in pennation angle during the 30% SOL EMG condition and a main effect of muscle at 60% SOL EMG (Fig. 3C,D). Specifically, at 30% SOL EMG there was less fascicle rotation in the knee-flexed than the knee-extended condition (KE, 5.5±4.0 deg; KF, 3.8±4.2 deg; *P*=0.024), regardless of the muscle. At 60% SOL EMG there was no effect of knee angle on fascicle rotation (*P*=0.148). Between muscle differences were observed such that the SOL and MG both had greater fascicle rotation than the LG at 30% SOL EMG (both *P*<0.001) and 60% SOL EMG (SOL, *P*=0.018; MG, *P*<0.001), regardless of knee angle.

#### Fascicle length

At both 30% and 60% SOL EMG, there were main effects of knee angle and muscle on fascicle shortening (Fig. 3E,F). During the knee-flexed condition, there was less fascicle shortening than during the knee-extended condition at both 30% SOL EMG (KE, −20.1±10.8%; KF, −13±8.6%; *P*<0.001) and at 60% SOL EMG (KE, −28.1±10.7%; KF, −21.6±11.4%; *P*=0.006), regardless of the muscle. The MG had greater fascicle shortening than the LG at both 30% SOL EMG (*P*=0.025) and 60% SOL EMG (*P*=0.002), with no differences between the MG and SOL (30% SOL EMG, *P*=0.063; 60% SOL EMG, *P*=0.109) or the LG and SOL (30% SOL EMG, *P*=1.00; 60% SOL EMG, *P*=0.608).

#### Time-varying difference in muscle thickness

**Fig. 4.**
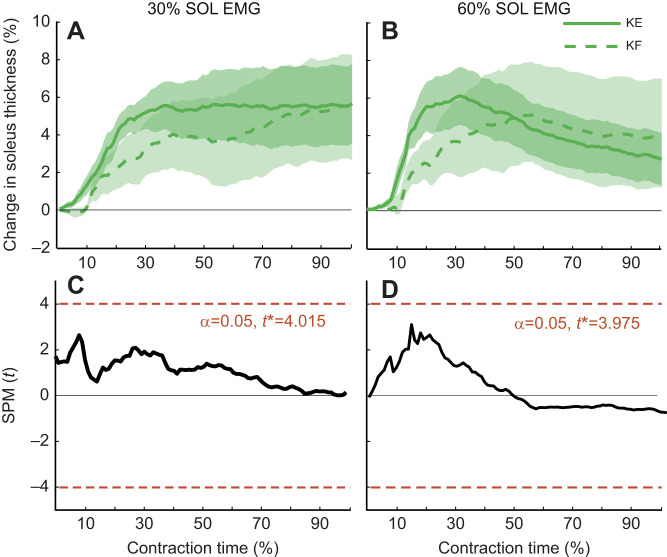
**Changing knee angle does not systematically influence the time-varying patterns of soleus (SOL) muscle shape change.** Change in SOL muscle thickness over the duration of the isometric contraction (normalized to resting thickness for each condition) at 30% (A) and 60% (B) maximum SOL EMG. The mean (*n*=11 participants) change in muscle thickness across all participants is indicated by the solid line for the knee-extended condition and the dashed line for the knee-flexed condition, with the standard error shown by the shaded regions. One-dimensional statistical parametric mapping (SPM) was used to determine whether time-varying SOL muscle thickness differed between knee extended (KE) and knee flexed (KF) conditions at 30% (C) and 60% (D) maximum SOL EMG using a two-tailed *t*-test with an alpha level (α) set to 0.05 and critical *t* (t*) indicated by the dashed red lines. The solid black lines display the *t*-statistic at each time point throughout the contraction time (%). Change in SOL muscle thickness was not significantly different between KE and KF at 30% or 60% of maximum SOL EMG.

**Fig. 5.**
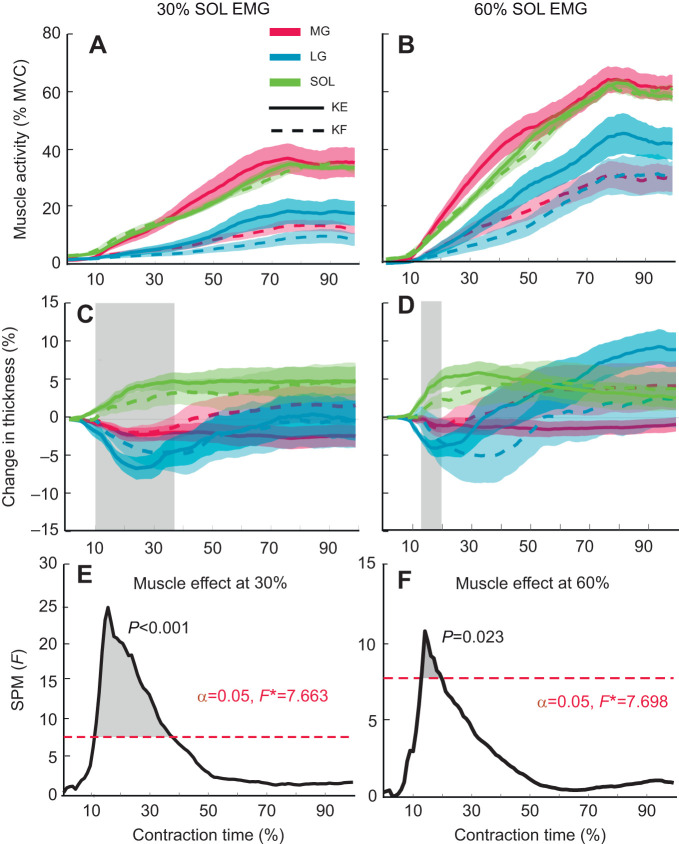
**Muscles of the triceps surae demonstrated different neuromechanical behaviours during the plantarflexion contraction.** Time-varying normalized muscle activity (A,B), change in muscle thickness (C,D) and SPM output (*F*-statistic) for the change in muscle thickness (E,F) at 30% (A,C,E) and 60% (B,D,F) maximum SOL EMG. Data shown as the average (mean) across all participants (*n*=11) for the knee extended (KE, solid line) and knee flexed (KF, dashed line) conditions with the standard error shown as the shaded area for the MG (red), LG (blue) and SOL (green). One-dimensional statistical parametric mapping was used to perform a two-way repeated measures ANOVA to determine the effect of knee angle and muscle on the change in thickness. There was an effect of muscle, but not knee angle, at 30% (E) and 60% (F) conditions with an alpha level (α) set to 0.05 and critical *F* (F*) indicated by the dashed red lines. The black line displays the *F*-statistic at each time point throughout the contraction. The grey shaded areas indicate time points of the contraction where there was a significant difference in the changes in thickness between muscles.

There was no difference in the time-varying changes in SOL muscle thickness between knee angles at 30% SOL EMG (critical *t*=4.02; maximum *t*=2.64) (Fig. 4A,C) or 60% SOL EMG (critical *t*=3.98; maximum *t*=3.04) (Fig. 4B,D). There was also no difference in time-varying thickness of the MG or LG between knee angles at 30% SOL EMG (critical *F*=14.90; maximum *F*=2.33) (Fig. 5C) or 60% SOL EMG (critical *F*=15.00; maximum *F*=2.29) (Fig. 5D). No interactions between knee angle and muscle were identified from the 30% SOL EMG (critical *F*=7.663; maximum *F*=4.29) or 60% SOL EMG (critical *F*=7.69; maximum *F*=5.35) conditions. However, there were differences in the thickness between muscles of the triceps surae during the force rise region of each contraction, such that SOL thickness increased while the gastrocnemii thickness decreased or remained relatively constant. These differences were observed between 10% and 36% (i.e. during the ramp phase) of the contraction time in the 30% SOL EMG condition (critical *F*=7.66; maximum *F*=24.39; *P*<0.001) (Fig. 5C,E), and between 13% and 20% of the contraction time for the 60% SOL EMG condition (critical *F*=7.69; maximum *F*= 10.73; *P*=0.023) (Fig. 5D,F).

## DISCUSSION

Here, we examined the influence of altered triceps surae contractile environment on changes in muscle shape during submaximal contractions. As expected, changes in knee angle altered the neuromechanical contributions of the gastrocnemii to ankle torque. Ankle plantarflexion contractions with a flexed knee angle were associated with lower gastrocnemii muscle activity and shorter muscle fascicle lengths. However, despite these changes in the external contractile environment, the SOL did not bulge more in thickness when the knee was flexed, as hypothesized. This was unexpected, given the ability of our experimental protocol to reduce gastrocnemii muscle activity by ∼50% whilst maintaining SOL activity in the knee-flexed posture. Our results demonstrated that patterns of muscle shape change (in the thickness direction) within the triceps surae varied considerably across participants, which could be related to differences in muscle mechanical properties or motor control strategies.

### Neuromechanical interactions between triceps surae muscles

Given the dynamic and transient changes in muscle thickness, we considered the magnitude as well as the temporal patterns of muscle thickness change in the triceps surae. A comparison of changes in thickness over time provide evidence of an interaction between muscles, particularly during the ramp phase of the isometric contractions where the activity levels, and thus forces, were low. When the SOL bulged in thickness, there was a simultaneous thinning that occurred in the MG and LG, consistent with results reported by Hodson-Tole and Lai (2019). To explore this further, we examined the relationship between time-varying thickness changes and muscle activity. We found that the LG activated later in the contraction in all conditions and the largest differences between SOL and gastrocnemii bulging coincided with the lowest levels of MG and LG activity. The SOL bulged in thickness initially, likely applying a transverse force to the passive LG, and in some conditions, the MG. Bulging of the SOL early in the contractions was associated with a simultaneous thinning of the LG until the later onset of LG activity, at which time the LG transitioned to bulging in thickness (Fig. 5A–D). Previous studies have demonstrated that when muscles are transversely loaded, they perform work against the applied load, particularly when loaded in the thickness direction (Ryan et al., 2019; Siebert et al., 2014; Stutzig et al., 2019). Muscle thickness in the MG remained relatively constant throughout the contraction, likely bulging in width to accommodate its muscle length changes. Given the anatomical arrangement of the triceps surae, width-wise expansion of the MG would also act to deform the LG in the width direction, and may further contribute to the LG's increase in thickness during the later phase of the contraction (Hodson-Tole and Lai, 2019; Kelp et al., 2021; Maganaris et al., 1998; Wakeling et al., 2011). Although this mechanism is supported by the group level data, there was not a uniform pattern of muscle thickness changes across participants, and the variability in bulging behaviour appeared to increase when the neuromechanical contributions of the gastrocnemii were reduced.

Interactions between muscles occur not only via bulging and transverse loading, but also through longitudinal strains produced by muscles sharing an aponeurosis (Finni et al., 2017, 2023). Epimuscular force transmission between muscles can occur via compression, as noted previously, or through shear and tensile forces. Previous *in vivo* studies revealed similar aponeurosis displacements between the SOL and gastrocnemii (MG: Bojsen-Moller et al., 2010; LG: Finni et al., 2017) during active muscle shortening, indicating epimuscular force transmission via tensile forces. Here, we find that the SOL underwent less fascicle shortening and rotation when the knee was flexed compared with extended (Fig. 3), despite similar levels of muscle activity. Shortening of the MG and LG muscle fascicles likely amplifies SOL owing to the opposing fascicle orientations (Fig. 1D) and epimuscular connections between adjacent aponeuroses. When the knee was flexed, the MG and LG underwent less fascicle shortening, thereby limiting the epimuscular tensile forces acting on the SOL, leading to less fascicle shortening and therefore smaller requirements for the muscle to bulge in thickness to displace its volume. The lack of bulging differences in the SOL between the knee-flexed and knee-extended positions may be, in part, due to a trade-off between the compressive and tensile epimuscular forces transmitted between the synergist muscles of the triceps surae (Maas and Sandercock, 2010). These results are consistent with others (Bojsen-Moller et al., 2010; Finni et al., 2017), demonstrating that altering the contractile environment (i.e. changing knee angle and muscle activities of surrounding muscles) influences muscle fascicle dynamics and therefore probably affects muscle gearing.

### Inter-individual variability in muscle behaviour

We observed a wide range of muscle bulging behaviours across participants, particularly in the MG and SOL, despite the constrained nature of the contractions. This range was even greater in the knee-flexed condition when the neuromechanical contributions of the gastrocnemii was low (Fig. S1). Given that muscle stiffness increases with muscle fascicle length (above slack length) and activation level (Raiteri et al., 2018, 2016), shorter fascicle lengths and lower activity in the MG and LG during the knee flexed conditions would likely lead to a lower gastrocnemii active muscle stiffness, and thus less resistance to transverse loading imposed by the bulging of neighboring muscles. By contrast, when the knee was extended, higher activity in surrounding muscles, and thus greater contributions of active muscle stiffness, should limit the range of deformations that any one muscle could undergo. This supports the idea that changing the contractile environment does influence muscle shape changes; however, the response of each muscle was different between participants. We considered if this variability in our data could be related to experimental noise or methodological error (e.g. probe location, orientation, movement), but if this were the case, we might expect the same amount of variability in the knee extended and knee flexed conditions, which we did not see. Some likely sources of this variability are differences in the passive mechanical properties of these muscles or to differences in the coordination strategy between the MG and LG across participants, yet the interactions between passive and active neuromechanical properties and their influence on *in vivo* muscle deformations remains largely unexplored.

The muscles within the triceps surae demonstrate regional differences in deformation. For example, magnetic resonance imaging of the MG during 20% contraction levels with an extended knee led to greater bulging in width and decreased thickness at the proximal region of the muscle, but increased thickness and decreased width in the distal region (Wakeling et al., 2020). The response of a muscle to transverse loading can be influenced by internal muscle properties, muscle activity level and the direction of external loading (Ryan et al., 2020). A potential explanation for the lack of differences in SOL thickness change within the altered contractile environment could be the interindividual differences in the distribution of activation across muscles of the triceps surae, which would in turn induce variability in the distribution of muscle mechanical properties across participants (Nordez and Hug, 2010). Numerous studies have demonstrated large inter-individual differences in the distribution of activation between the gastrocnemii during isometric contractions (Crouzier et al., 2018), walking (Ahn et al., 2011; Crouzier et al., 2019; Hamard et al., 2021) and pedalling (Crouzier et al., 2019; Hug et al., 2008, 2010). To explore if the variability in thickness change patterns could be explained by individual differences in the distribution of muscle activity, we examined whether the difference in the distribution of gastrocnemii muscle activity (estimated using equation 3 in Crouzier et al., 2018) between the knee-extended and knee-flexed conditions was associated with the difference in SOL thickness between these same conditions. Exploratory analysis demonstrated no relationship between SOL bulging and the distribution of gastrocnemii activity (difference between knee-extended and knee-flexed conditions) (*R*^2^=0.01, *P*=0.897), suggesting that the distribution of activation alone cannot explain the variability in SOL bulging across participants. However, this analysis was conducted at one time point (the plateau region) of the contraction. We consistently observed the SOL and LG to display opposite bulging behaviour (i.e. one muscle gets thinner while other gets thicker) at the beginning of the contraction. However, the timing and magnitude of this interaction varied between participants as demonstrated across two participants (Fig. S2). It is difficult to determine why some individuals have specific bulging patterns over others; however, it is likely there is a time-dependent influence of activation and surrounding muscle bulging, as well as differences in muscle mechanical properties, which together contribute to these varied behaviours.

### Functional consequences of an altered contractile environment

We expected greater torque in the knee-extended condition compared with the knee-flexed conditions, as there is a greater contribution of the MG and LG. However, at 30% SOL EMG, we found no difference in ankle plantarflexion torque between the knee extended and knee flexed conditions and at 60% SOL EMG, torque was ∼19% lower in the knee-flexed conditions (Table 1). Considering MG and LG activity was nearly 50% lower when the knee was flexed compared with extended, the magnitude of torque reduction was less than expected from changes in EMG alone. This may be related to other factors that influence a muscles force production (i.e. the force–length relationship), or to interactions between muscles. For example, there is evidence that the transverse loading of a muscle, due to the bulging of other muscles within a compartment, has the potential to both reduce (de Brito Fontana et al., 2020; Ryan et al., 2020) or increase (Ryan et al., 2020) the amount of force along the muscles line of action. This has previously been described *in silico* whereby contracting muscles with higher pennation angles (>20 deg) and longer fascicle lengths have greater longitudinal force when transversely loaded in the thickness direction, owing to the redistribution of strain-energy potentials from volumetric compression and the muscles’ base materials (i.e. connective tissue) (Ryan et al., 2020; Wakeling et al., 2020). However, when muscles with shorter lengths were loaded in the thickness direction or muscles at different lengths are loaded in the width direction, there were reductions in longitudinal muscle force (Ryan et al., 2020).

The limited decreases in ankle torque observed here, despite large reductions in gastrocnemii activity between the knee-extended and knee-flexed conditions, may be related to the complex interaction of loading direction, muscle lengths, pennation angles and activity between the triceps surae muscles. There was no difference in SOL resting fascicle length or pennation angle between knee-extended and knee-flexed conditions, whereas the MG and LG both had shorter fascicles in the knee-flexed condition. All three muscles had a greater range of bulging behaviours in the knee-flexed condition. This may increase the transverse loading on the SOL and may enhance SOL force output in the longitudinal direction as described by Ryan et al. (2020), contributing a proportion of the torque lost owing to lower gastrocnemii activity. However, when the knee was extended, greater activation and therefore greater stiffness (Finni et al., 2017; Raiteri et al., 2018) across all the gastrocnemii may result in increased pressure within the compartment of the lower leg and transverse loading on triceps surae muscles (Maas, 2019). This transverse loading may act to reduce the longitudinal force output of the muscles when they are activated simultaneously (de Brito Fontana et al., 2020; Siebert et al., 2014), as well as limit the range of deformations the muscles could undergo (de Brito Fontana et al., 2020; Siebert et al., 2014). However, the influence of and mechanisms linking intramuscular pressure, transverse loading and force production remain inconclusive. Future experiments that incorporate 3D muscle imaging or employ a combination of *in vivo* and *ex vivo* techniques, might reveal a more detailed picture of how muscles respond to transverse loading from neighbouring muscles.

### Considerations and opportunities

In this study, there are limitations that should be considered, presenting opportunities for future research to build upon. We characterized muscle shape changes in two dimensions (accounting only for changes in thickness), although we know that muscles bulge in 3D (thickness and width) and that there may be a ‘shearing’ of the aponeuroses that occurs during a contraction which cannot be visualized. Therefore, changes in muscle thickness may be masked because of muscle ‘rotations’. Future experiments, utilizing tools such as fluoromicrometry (Camp et al., 2016) to track not just the length, width and thickness, but also the shearing of the aponeuroses may help us understand to what degree we under- or overestimate changes in thickness that are measured using 2D ultrasound. *In vivo* 3D imaging approaches may also enable the visualization of the interactions and timing of muscle bulging within a compartment (Wakeling et al., 2020). It should also be noted that ultrasound images of the SOL were collected deep to either gastrocnemii, depending on which muscle allowed for a better image quality. Seven participants' SOL were imaged deep to the MG and four deep to to the LG. In this study, we assumed that the SOL would have similar bulging behaviours in regions as deep as both gastrocnemii; however, regional differences in muscle shape change are known to occur and may account for some of the variability observed within this study (Hodgson et al., 2006; Wakeling et al., 2020).

### Conclusions

Our muscles undergo constantly undergo changes in their contractile environment whether from our immediate movements or from adaptations to our muscle properties (i.e. architecture, intramuscular fat and stiffness) (Kelp et al., 2023) over time (i.e. ageing and disuse) (Pinel et al., 2021; Randhawa and Wakeling, 2013). For example, lower force generating capacity and greater intramuscular fat were linked to reduced shape changes in the gastrocnemii in less active older adults, which may impact how muscles interact within a compartment. Understanding how skeletal muscles deform during contraction has implications for the mechanics and energetics of movement (Ryan et al., 2020). Here, we found that altering the contractile environment of the triceps surae by changing the knee angle led to reductions in the neuromechanical contribution of the gastrocnemii but no accompanying changes in soleus bulging. Despite the controlled nature of the ramped isometric plantarflexion contractions whereby SOL muscle activity was maintained and gastrocnemii activity was reduced, there were a variety of different triceps surae bulging strategies demonstrated across participants. The large inter-individual variability in shape change patterns may be related to differences in muscle activity and mechanical properties between participants and across muscles. Future studies that combine *in vivo* and *ex vivo* measurements of muscle deformation across scales from single isolated muscles to groups of synergist muscles could provide exciting insights, with implications for understanding force production in other scenarios such as during compression (i.e. garments, sitting) (Stutzig et al., 2019) or aging, where sarcopenic changes can happen at different rates between muscles (Pinel et al., 2021).

## Supplementary Material

10.1242/jexbio.248118_sup1 Supplementary information
