# Supplementary material for: Effects of altered contractile environment on muscle shape change in the human triceps surae
Source: J Exp Biol. 2024 Dec 2;227(23):jeb248118. doi: 10.1242/jeb.248118 (PMC11658685; doi:10.1242/jeb.248118)
Supplement: Supplementary information [file jexbio-227-248118-s1.pdf]

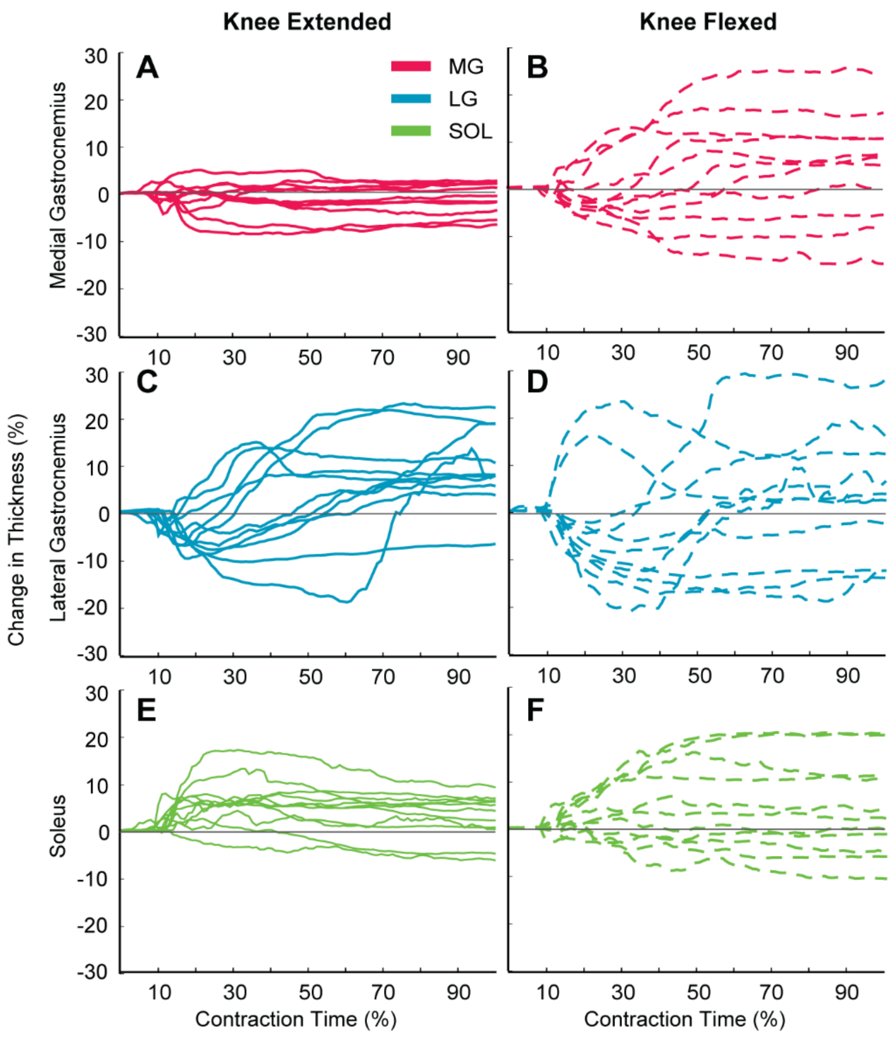

Fig. S1. Patterns of muscle shape changes varied between individuals. Time-varying changes in muscle thickness shown for each participant during the 60% maximum SOL EMG contraction for the medial gastrocnemius (MG: red; A, B), lateral gastrocnemius (LG: blue; C, D) and soleus (SOL: green; E, F) for each knee angle with the knee extended shown in solid lines (A, C, E) and knee flexed shown in dashed lines (B,D,F). The change in thickness was normalized to resting muscle thickness for each participant at each condition.

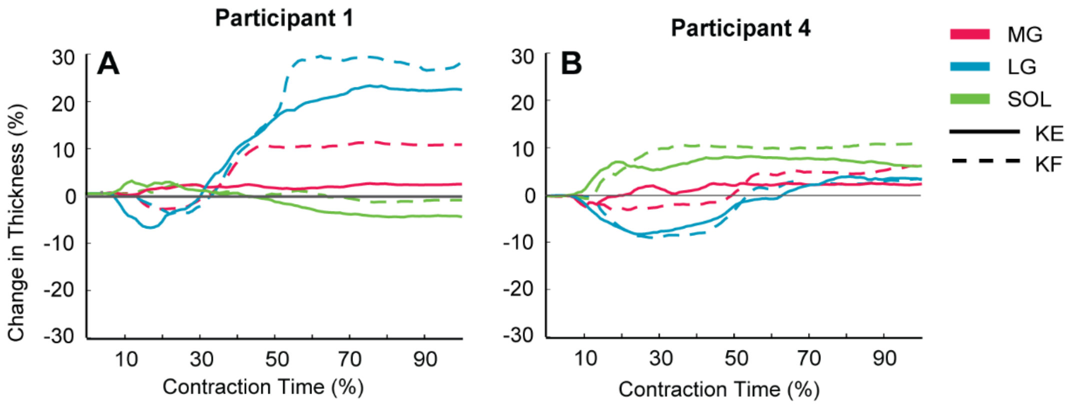

Fig. S2. Example of individual muscle shape change patterns for two participants (A; B) during the 60% maximum SOL EMG contraction for the medial gastrocnemius (MG, red), lateral gastrocnemius (LG, blue) and soleus (SOL, green). The knee extended conditions are shown as solid lines and knee flexed as dashed lines. Both participants demonstrated opposite SOL and LG bulging at the beginning of the contraction, with the SOL getting thicker and LG getting thinner, however in Participant 1 (A) the SOL bulges less in thickness at the beginning of the contraction and later thins or remains constant, regardless of knee angle. Participant 2 (B) SOL displayed greater bulging in thickness which remained for the duration of the contraction, while the LG transitioned from getting thinner to thicker when the SOL maintained a constant thickness.

Journal of Experimental Biology • Supplementary information

Table S1. Summary of statistical results from the linear mixed effects models including the full models as well as the Tukey Post hocs.

Key: condition: knee angle position, KE: knee extended, KF: knee flexed, MG: medial gastrocnemius, LG: lateral gastrocnemius, SOL: soleus

**Resting Fascicle Length**			
**full model**	lme(FL_mm~ condition *muscle, random = subject)		
	condition	$F_{1,50}$= 13.9184	p<0.001*
	muscle	$F_{2,50}$= 42.9145	p<0.001*
	condition * muscle	$F_{2,50}$= 9.1315	p<0.001*
**MG Tukey post-hoc**	KE > KF		p<0.001*
**LG Tukey post-hoc**	KE > KF		p<0.001*
**SOL Tukey post-hoc**	KE < KF		p= 0.007*

**Resting Pennation Angle**			
**full model**	lme(PA_deg~ condition *muscle, random = subject)		
	condition	$F_{1,50}$= 0.8433	p=0.3629
	muscle	$F_{2,50}$= 75.8939	p<0.001*
	condition * muscle	$F_{2,50}$= 5.9939	p=0.0046*
**MG Tukey post-hoc**	KE > KF		p=0.0045*
**LG Tukey post-hoc**	KE = KF		p=0.116
**SOL Tukey post-hoc**	KE < KF		p= 0.0042*

**Resting Muscle Thickness**			
**full model**	lme(TH_mm~ condition *muscle, random = subject)		
	condition	$F_{1,50}$= 10.3733	p=0.0022*
	muscle	$F_{2,50}$= 30.3652	p<0.001*
	condition * muscle	$F_{2,50}$= 2.5769	p=0.0861
**MG Tukey post-hoc**	KE > KF		p<0.001*
**LG Tukey post-hoc**	KE > KF		p<0.001*
**SOL Tukey post-hoc**	KE = KF		p= 0.989

**Muscle Activity at 30% SOL EMG**			
**full model**	lme(emg30 ~ condition*muscle, random = subject)		
	condition	$F_{1,50}$= 21.85088	p<0.001*
	muscle	$F_{2,50}$= 30.07992	p<0.001*
	condition*muscle	$F_{2,50}$= 8.93917	p<0.001*
**MG Tukey post-hoc**	KE > KF		p<0.001*
**LG Tukey post-hoc**	KE > KF		p=0.013*
**SOL Tukey post-hoc**	KE = KF		p=0.841
**Muscle Activity at 60% SOL EMG**			
**full model**	lme(emg60 ~ condition*muscle, random = subject)		
	condition	$F_{1,50}$= 24.59035	p<0.001*
	muscle	$F_{2,50}$= 16.83296	p<0.001*
	condition*muscle	$F_{2,50}$= 7.76685	p=0.0012*
**MG Tukey post-hoc**	KE > KF		p<0.001*
**LG Tukey post-hoc**	KE > KF		p=0.024*
**SOL Tukey post-hoc**	KE = KF		p=0.0703
**Change in Muscle Thickness during Hold at 30% SOL EMG**			
**full model**	lme(THc_per30~ condition*muscle, random = subject)		
	condition	$F_{1,50}$= 0.2632605	p=0.6101
	muscle	$F_{2,50}$= 2.6150432	p=0.0831
	condition*muscle	$F_{2,50}$= 0.6353424	p=0.5340
**Change in Muscle Thickness during Hold at 60% SOL EMG**			
**full model**	lme(THc_per60~ condition*muscle, random = subject)		
	condition	$F_{1,50}$= 0.014939	p=0.9032
	muscle	$F_{2,50}$= 1.127663	p=0.3319
	condition*muscle	$F_{2,50}$= 2.806316	p=0.0700

Journal of Experimental Biology • Supplementary information

**Change in Pennation Angle during Hold at 30% SOL EMG**			
**full model**	lme(Pac_deg30~ condition*muscle, random = subject)		
	condition	$F_{1,50}$= 5.395617	p=0.0243*
	muscle	$F_{2,50}$= 9.476668	p=0.0003*
	condition*muscle	$F_{2,50}$= 0.939517	p=0.3976
**Muscle Tukey post-hoc**	LG<MG&SOL, MG=SOL		p<0.001*, p<0.001*, p=0.6448
**Change in Pennation Angle during Hold at 60% SOL EMG**			
**full model**	lme(PAc_deg60~ condition*muscle, random = subject)		
	condition	$F_{1,50}$= 2.16358	p=0.1476
	muscle	$F_{2,50}$= 10.19512	p<0.001*
	condition*muscle	$F_{2,50}$= 0.50525	p=0.6064
**Muscle Tukey post-hoc**	LG<MG&SOL, MG=SOL		p<0.001*, p=0.0177*, p=0.1681
**Change in Fascicle Length during Hold at 30% SOL EMG**			
**full model**	lme(FLc_per30~ condition*muscle, random = subject)		
	condition	$F_{1,50}$= 14.69272	p<0.001*
	muscle	$F_{2,50}$= 3.78188	p=0.0295*
	condition*muscle	$F_{2,50}$= 0.23051	p=0.7950
**Muscle Tukey post-hoc**	LG<MG, LG=SOL, MG=SOL		p=0.0253*, p=0.0634, p=1.000
**Change in Fascicle Length during Hold at 60% SOL EMG**			
**full model**	lme(FLc_per60~ condition*muscle, random = subject)		
	condition	$F_{1,50}$= 8.15081	p=0.0063*
	muscle	$F_{2,50}$= 5.46403	p=0.0071*
	condition*muscle	$F_{2,50}$= 0.97649	p=0.3837
**Muscle Tukey post-hoc**	LG<MG, LG=SOL, MG=SOL		p=0.0023*, p=0.10877, p=0.6078
